# A Novel Strategy to Regulate 1-Deoxynojirimycin Production Based on Its Biosynthetic Pathway in *Streptomyces lavendulae*

**DOI:** 10.3389/fmicb.2019.01968

**Published:** 2019-08-22

**Authors:** Hao Wu, Ye Guo, Lei Chen, Guiguang Chen, Zhiqun Liang

**Affiliations:** State Key Laboratory for Conservation and Utilization of Subtropical Agro-Bioresources, Guangxi Microorganisms and Enzyme Research Center of Engineering Technology, College of Life Science and Technology, Guangxi University, Nanning, China

**Keywords:** 1-deoxynojirimycin, biosynthetic pathway, *Streptomyces lavendulae*, precursors, metabolism inhibitors

## Abstract

This study characterized the biosynthetic pathway of the secondary metabolite 1-deoxynojirimycin (DNJ) from *Streptomyces lavendulae*. The results revealed that glucose was a preferable precursor for DNJ synthesis, and its carbon skeleton underwent a C2-N-C6 cyclization reaction during synthesis. The biosynthetic pathway was related to the glycolysis pathway, and started from fructose-6-phosphate, and involved amination, dephosphorylation, oxidation, cyclization, dehydration, and reduction reaction steps, yielding DNJ. Then, based on clarified biosynthetic pathway information, precursors, analogs, and metabolism inhibitors were used as novel regulators to enhance the production of DNJ. The results demonstrated that the titer of DNJ could reach 296.56 mg/L, which was 3.3-fold higher than that of a control group (90 mg/L) when sodium citrate (0 h, 5 g/L), sorbose (0 h, 1 g/L), iodoacetic acid (20 h, 50 mg/L), and glucose (26 h, 7 g/L) were added during the fermentation process. This study provides a new understanding of the biosynthetic pathway of DNJ, and also provides an efficient strategy to regulate the production of DNJ based on this biosynthetic pathway, which is a new perspective for the regulation of other secondary metabolites.

## Introduction

1-Deoxynojirimycin (DNJ), a type of alkaloid secondary metabolite, was first isolated from mulberry trees by Yagi et al. (1976). Its structure is very similar to glucose, in which the oxygen atom of the pyranose ring is replaced by a nitrogen atom (Nakagawa et al., 2007). Therefore, as a saccharide decoy compound, it exerts significant α-glucosidase inhibitory activity and has been employed as food- and drug-grade DNJ for therapeutic purposes, such as anti-diabetic, anti-viral, and anti-HIV applications (Hu et al., 2017).

In addition to higher plants such as mulberry and dayflower (Shibano et al., 2004), DNJ can also be synthesized by certain microbes, such as *Bacillus* sp. (Onose et al., 2013) and *Streptomyces* sp. (Paek et al., 1997). However, DNJ content varies greatly depending on the source. A previous study reported that the DNJ content in mulberry products was as low as 0.1% (Kimura et al., 2007). Furthermore, the DNJ content in mulberry is affected by many factors, such as the growing season, the age of the leaves, and the climate, which makes DNJ levels extremely variable (Hu et al., 2013). It was reported that the titer of DNJ in *B. amyloliquefaciens* AS385 could reach 460 mg/L when cultured at 37°C with reciprocal shaking at 90 rpm for 5 days in a 500 mL shaking flask containing 50 mL of 40 g/L of soybean peptone supplemented with 50 g/L of sorbitol (Onose et al., 2013). The titer of DNJ in *Streptomyces* sp. SID9135 could reach 640 mg/L using an optimum medium composed of 25 g/L of lactose, 20 g/L of soybean meal, 4 g/L of yeast extract, 2 g/L of CaCO_3_, and 0.05 g/L of MgSO_4_^∙^7H_2_O at 29°C with an aeration rate of 1 vvm in a jar fermentor (Paek et al., 1997). Therefore, microbial DNJ production has received significant attention and interest, because microorganisms demonstrate rapid growth, controllable product yield, higher DNJ content, and a lower cost of cultivation. In the past few decades, many regulatory strategies for the production of DNJ have been employed, such as medium optimization (Wei et al., 2011), physical and chemical mutagenesis (Ezure et al., 1985), and culture condition control (dissolved oxygen, pH) (Kojima et al., 1995). Various microorganisms isolated from Korean traditional fermented food have been used to enhance DNJ content in mulberry leaf, including lactic acid bacteria, yeast, and *Bacillus* (Jeong et al., 2014).

The biosynthetic pathway for DNJ has been reported in some species. For example, key genes involved in DNJ biosynthesis in mulberry (*Morus alba* L.) have been identified through transcriptome analysis, and the biosynthetic pathway starting from aspartate has been outlined (Wang et al., 2018). In addition, Hardick et al. (1992) and Hardick and Hutchinson (1993) demonstrated about 26 years ago that glucose was a precursor for the synthesis of DNJ in *B. subtilis* and *S. subrutilus* in precursor feeding experiments using isotope-labeled glucose. During the synthesis process, DNJ was shown to be generated through a C2-N-C6 cyclization reaction as demonstrated by the isotope-labeling at the ^13^C1 of glucose ending at the ^13^C6 of DNJ. This reaction occurred in both *B. subtilis* and *S. subrutilus*. However, Shibano et al. (2004) suggested a very interesting biosynthetic route in *Commelina communis*, which is distinct from that in *B. subtilis* and *S. subrutilus*. It was found that DNJ from *C. communis* was formed via a C1-N-C5 cyclization reaction, as verified by the findings that the isotope-labeling at the ^13^C1 of glucose ended at the ^13^C1 of DNJ. Given the findings of the above studies, it can be suggested that the synthetic routes of DNJ are diverse, with differences between microorganisms and plants, and even plants themselves (*M. alba* L. and *C. communis*).

*Streptomyces lavendulae*, another mainstream DNJ-producing strain, has been reported in many studies (Ezure et al., 1985; Kojima et al., 1995; Wei et al., 2011). However, the biosynthetic pathway for DNJ in *S. lavendulae* is unknown, as is its similarity to that in *B. subtilis* or *S. subrutilus*. This lack of information has greatly restricted the development of *S. lavendulae* as a DNJ-producing strain, because the DNJ biosynthetic pathway is crucial in regulating the production of DNJ. There are no recent studies investigating the regulation of DNJ production using feeding experiments with specific inhibitors and precursors from biosynthetic pathways. Therefore, characterization of the biosynthetic pathway of DNJ in *S. lavendulae* is important and significant. Furthermore, the elucidation of the DNJ biosynthetic pathway would be useful in regulating DNJ production.

Therefore, the current study attempted to elucidate the biosynthetic pathway of DNJ in *S. lavendulae* through precursor feeding experiments, analyzing carbon skeleton conversion using isotope-labeled glucose, and identifying related metabolic intermediates through hydrophilic interaction chromatography with tandem mass spectrometry (HILIC-MS/MS) techniques. Then, based on known biosynthetic pathway information, a novel strategy was designed to enhance the production of DNJ by feeding biosynthetic precursors, metabolic intermediate analogs, and specific metabolism inhibitors. The current study not only reveals novel information pertaining to the biosynthetic pathway of DNJ, but also provides an efficient strategy to regulate the production of DNJ, which may be relevant for the regulation of other secondary metabolites.

## Materials and Methods

### Materials

D-[1-^13^C] and D-[2-^13^C] glucose was purchased from Cambridge Isotope Laboratories, Inc., United States, and the ^13^C abundance was more than 99%, whereas 9-Fluorenylmethyl chloroformate (FMOC-Cl) (>99%) was obtained from Aladdin company (Shanghai), and 2-Amino-2-deoxy-D-mannitol (ADM) was chemically synthesized by Ruizhi Chemical Company (Chengdu, China). Standard DNJ was purchased from Wako (Osaka, Japan). Other chemical reagents were of analytical grade.

### Microorganisms, Medium and Culture Conditions

*Streptomyces lavendulae* UN-8 from the China Center for Type Culture Collection (CCTCC M 2015512) was used in our study (Wu et al., 2017). The basal fermentation medium (BFM) was composed of (per liter) yeast extract 10 g, glucose 5 g, K_2_HPO_4_^∙^3H_2_O 0.5 g, KNO_3_ 0.5 g, NaCl 0.5 g, and MgSO_4_^∙^7H_2_O 0.5 g. The seed medium was composed of BFM in which glucose (5 g/L) was replaced with a soluble starch (20 g/L). All the media were adjusted to pH 7.30 ± 0.10 using 1 M NaOH or 1 M HCl and autoclaved at 115°C for 30 min. All culture conditions were set at 30°C and 180 rpm.

### Precursor Analysis for DNJ Synthesis

To determine a preferable carbon source to provide a carbon skeleton for DNJ synthesis and energy for cell growth, different carbon sources including glucose, fructose, galactose, lactose, maltose, sucrose, or soluble starch, were added into BFM at 24 h to evaluate their effect on the production of DNJ. Briefly, exponential growth phase cultures (1.0 mL) were inoculated into 50 mL of fresh BFM in a shake flask (250 mL) and fermented for approximately 24 h. Then, potential precursors (5 g/L) were added separately into the BFM, and it was cultivated for approximately 48 h under the same conditions. The cells were removed 3 days later through centrifugation at 12,000 rpm for 15 min, and the supernatant containing DNJ was assessed using a reversed-phase high-performance liquid chromatography (RP-HPLC) method (Kim et al., 2003). BFM without an additionally added carbon source at 24 h served as a control group.

### Metabolic Intermediate Identification Using a HILIC-MS/MS Technique

Samples were prepared in accordance with the method reported by Onose et al. (2013). In brief, lyophilized cells (1 g) were mixed with 20 mL of ethanol (70%, v/v) under ultrasonic treatment for 40 min. Subsequently, cell debris was removed through centrifugation and the obtained supernatant was filtered using a 0.22 μm millipore membrane. A 1.0 μL aliquot was injected into a HILIC-MS/MS system to analyze metabolic intermediates and related compounds.

The HILIC-MS/MS system consisted of an Ultra Performance Liquid Chromatography (UPLC) and a XEVO G2-S QTOF-MS/MS (Waters, United States). A 1.0 μL aliquot of a cell extract was separated using an ACQUITY UPLC BEH HILIC column (2.1 × 100 mm, 1.7 μm, Waters). The chromatography conditions were as follows: column temperature, 35°C; flow rate, 0.3 mL min^–1^. The column was isocratically eluted with mobile phase solvent A (99.5%) and solvent B (0.5%), where solvent A consisted of water containing 0.1% formic acid, and solvent B consisted of acetonitrile containing 0.1% formic acid. Subsequently, separated DNJ and metabolic intermediates were further identified through MS/MS with multiple reaction monitoring for the transition of the parent ions to the product ions. The MS/MS parameters (e.g., collision energy) were optimized in advance using standard DNJ and ADM under positive ion electrospray ionization.

### Isotope-Labeling Glucose Experiment

To clarify the origin and conversion of the carbon skeleton atoms in DNJ synthesized in *S. lavendulae*, labeled D-[1-^13^C] or -[2-^13^C] glucose (1 g, accounts for 10% of total glucose) was added into the BFM before inoculation. After cultivation for 3 days, the DNJ was purified through anion (201 × 4) and cation (001 × 7) exchange resin chromatography (unpublished work), and further characterized through ^13^C nuclear magnetic resonance (NMR) spectra.

### Effect of Different Metabolism Inhibitors on the Production of DNJ

Different concentrations of the mevalonate pathway inhibitor simvastatin (Buhaescu and Izzedine, 2007), the shikimate pathway inhibitor edetic acid (EDTA) (Sun et al., 2009), the Embden-Meyerhof-Parnas (EMP) pathway inhibitors iodoacetic acid (Wang et al., 2002) and sodium citrate (Liu et al., 2004), the hexose monophosphate pathway (HMP) inhibitor sodium phosphate (Yu and Pan, 1996), and the tricarboxylic acid cycle (TCA) inhibitor sodium malonate (Zeng et al., 2019) were added into the BFM at different time to evaluate their contribution to the production of DNJ. Detailed parameters concerning the use of these compounds, such as addition time and concentrations, are shown in Supplementary Table S1.

### Effect of Precursor and Intermediate Analogs on the Production of DNJ

Precursor analogs (mannose, sorbose) and intermediate analogs (mannitol, sorbitol, rhamnose) were added into BFM at 0 h to investigate their effect on the production of DNJ. The concentrations of these compounds ranged from 1 to 4 g/L.

### Effect of Precursor Glucose on the Production of DNJ

Different concentrations of precursor glucose ranging from 0.5 to 8 g/L were added into BFM at 24 h to evaluate their effect on the production of DNJ.

### Strategy Designed for the Production of DNJ

Based on the above single factor experiment results, factors that were highly significant for the production of DNJ, such as the EMP pathway inhibitors iodoacetic acid and sodium citrate, the precursor analogs sorbose and precursor glucose, were further selected to conduct an orthogonal experiment. An orthogonal L_27_(3^5^) test design (Menon et al., 2013) was used to optimize the production of DNJ in 250 mL shake flasks with the help of Minitab 17 Statistical software. A total of 27 fermentation experiments were performed using concentrations of supplemented glucose of 0.250, 0.300, and 0.350 g, time of glucose supplementation of 24, 26, and 28 h, concentrations of sorbose of 0.050, 0.075, and 0.100 g, and time of iodoacetic acid supplementation of 16, 18, and 20 h. The base medium was BFM containing sodium citrate (5 g/L). The concentration of supplemented iodoacetic acid was fixed at 50 mg/L. The time of sorbose addition was fixed at 0 h. Detailed information about the experimental conditions for the production of DNJ is shown in Supplementary Table S2.

### Analytical Method

The growth of *S. lavendulae* UN-8 was determined by assessing the dry cell weight (DCW). In brief, 50 mL of fermentation culture was processed through air pump filtration. After washing with distilled water, the separated mycelia were dried at 105°C to a constant weight. The amount of DNJ was determined by RP-HPLC coupled with a fluorescence detector (Kim et al., 2003). Briefly, 10 μL of sample and 10 μL of potassium borate buffer (0.4 M, pH 8.5) were mixed in a 1.5-mL microtube. Then 20 μL of FMOC-Cl (5 mM) dissolved in acetonitrile was added to the system and incubated at 20°C for 20 min. Subsequently, 10 μL of glycine (0.1 M) was added to terminate the reaction by quenching the remaining FMOC-Cl. Finally, 950 μL of aqueous acetic acid (0.1%, v/v) was added to the system to stabilize the FMOC-DNJ. Then, the amount of DNJ was analyzed using an Agilent Eclipse Plus C18 (4.6 mm × 250 mm, 5 μm) equilibrated with mobile phase (acetonitrile- aqueous acetic acid (0.1%, v/v), 50:50, v/v) at 25°C. A 10 μL aliquot treated through millipore filtration (0.45 μm) was injected and the flow rate was adjusted to 1 mL/min. The concentration of glucose was detected using a glucose oxidase and H_2_O_2_ electrode biosensor (SBA-40D, Shandong Academy of Sciences, China) (Pan et al., 2019).

## Results

### Characterization of the Biosynthetic Pathway of DNJ

#### Precursor Identification for DNJ Synthesis

**FIGURE 1:**
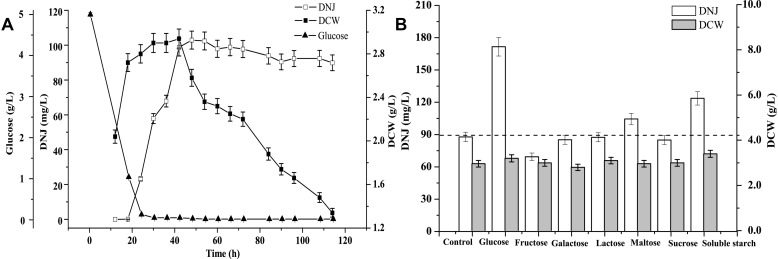
**(A)** Time course curves of DNJ content, biomass of UN-8, residual glucose during fermentation process. **(B)** The effect of adding different carbon source compounds at 24 h on the production of DNJ and biomass of UN-8.

Before identifying precursors for DNJ synthesis, a time course of the growth of UN-8, the titer of DNJ, and the concentration of residual glucose were assessed during whole fermentation. As shown in Figure 1A, the maximum DCW of UN-8 occurred at approximately 30 h, and the concentration of residual glucose had been completely consumed at approximately 24 h, while the maximum titer of DNJ was achieved at approximately 42 h. Thereafter, the DCW of UN-8 decreased dramatically because of an energy shortage, and the titer of DNJ was almost constant. The addition time of the different carbon sources was determined at the end of the logarithmic phase (24 h). The results are shown in Figure 1B. The carbon sources had a similar effect on the growth of UN-8 when compared with the control group. However, it was evident that the titer of DNJ could be increased by glucose, maltose, and soluble starch, at 1.95-fold, 1.19-fold, and 1.41-fold higher than that of the control group, respectively. In contrast, the other four carbon sources did not facilitate the production of DNJ. Therefore, it was reasonable to conclude that glucose was a preferable precursor for *S. lavendulae* UN-8 to synthesize DNJ.

### Metabolic Intermediates Identified Using HILIC-MS/MS

**FIGURE 2:**
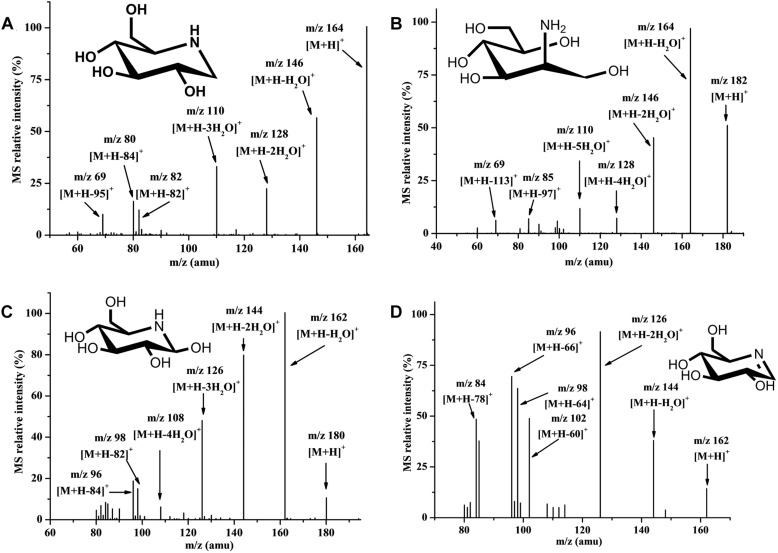
Secondary mass spectrum in positive ion mode. **(A)** Nojirimycin dehydrate. **(B)** 1-Deoxynorimycin (DNJ). **(C)** Nojirimycin (NJ). **(D)** 2-Amino-2-deoxy-D-mannitol (ADM).

It was difficult to isolate all intermediate compounds that were related to the synthesis of DNJ through traditional separation methods from cell extracts, and further identify their structures and molecular weight through NMR coupled with mass spectra (MS) techniques. The HILIC-MS/MS technique is effective for the analysis and identification of polar metabolic intermediate compounds because of its accuracy and it does not require a large sample volume (Onose et al., 2013). Using this technique, metabolic intermediate compounds related to the synthesis of DNJ such as nojirimycin (NJ) dehydrate (m/z 162) (Figure 2A), DNJ (m/z 164) (Figure 2B), NJ (m/z 180) (Figure 2C), and ADM (m/z 182) (Figure 2D), were successfully isolated, detected, and further identified based on the MS and MS/MS data given for chemically synthesized ADM or commercially available DNJ or literature containing NJ compound information (Onose et al., 2013), revealing their first existence in *S. lavendulae*. The secondary MS of these four compounds demonstrated a similar product ion fragment, which indicated similar structures among them. For example, NJ dehydrate (m/z 162) and NJ (m/z 180) had m/z 144 [M+H-18]^+^ or [M+H-36]^+^, m/z 126 [M+H-36]^+^ or [M+H-54]^+^, m/z 98 [M+H-64]^+^ or [M+H-82]^+^, m/z 96 [M+H-66]^+^ or [M+H-84]^+^ product ion fragments, respectively. DNJ (m/z 164) and ADM (m/z 182) had m/z 146 [M+H-18]^+^ or [M+H-36]^+^, m/z 128 [M+H-36]^+^ or [M+H-54]^+^, m/z 110 [M+H-54]^+^ or [M+H-72]^+^, m/z 69 [M+H-95]^+^ or [M+H-113]^+^ product ion fragments, respectively.

### Analysis Transformation of Carbon Skeleton Atoms Using Isotope-Labeled Glucose and NMR Spectroscopy

**FIGURE 3:**
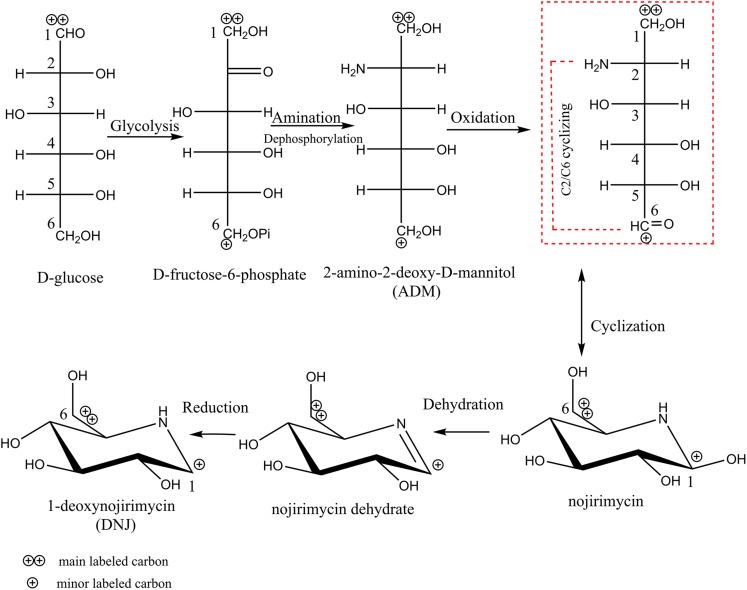
The schematic flow of biosynthetic pathway of DNJ in *S. lavendulae.*

It has been clarified above that glucose is a DNJ precursor, which provides a carbon skeleton for *S. lavendulae* UN-8 to synthesize DNJ. However, it is still unclear how the structure of the carbon skeleton of glucose changed during the synthesis process. The results of [1-^13^C] and [2-^13^C] labeled glucose experiments are shown in Supplementary Figure S1. When the BFM contained [1-^13^C] labeled glucose, the atomic abundance of glucose ^13^C1 finally accumulated on the ^13^C6 atom of DNJ (Supplementary Figure S1B) in contrast with the control (Supplementary Figure S1A). When the BFM contained [2-^13^C] labeled glucose, the atomic abundance of glucose ^13^C2 finally accumulated on the ^13^C5 atom of DNJ (Supplementary Figure S1C). This indicated that the glucose carbon skeleton underwent a C2-N-C6 cyclization reaction during the synthesis of DNJ, which is shown in Figure 3 (the red box compound).

Based on the above information concerning the glucose precursor, metabolic intermediate compounds, and the transformation of the carbon skeleton of glucose, the biosynthetic pathway of DNJ in *S. lavendulae* UN-8 can be inferred, and this is shown in Figure 3. First, glucose was converted to fructose-6-phosphate through the glycolysis pathway. Then, amination at the C2 position and dephosphorylation at the C6 position of fructose-6-phosphate produced an ADM intermediate. Subsequently, C6-OH in ADM would be further oxidized and form a 6-oxo species transient state that would quickly cyclize through C2-N-C6 to NJ. Finally, DNJ was generated after losing the 1-OH in NJ and the reduction of the NJ dehydrate intermediate.

### Regulation of the Production of DNJ Based on Its Biosynthetic Pathway

**FIGURE 4:**
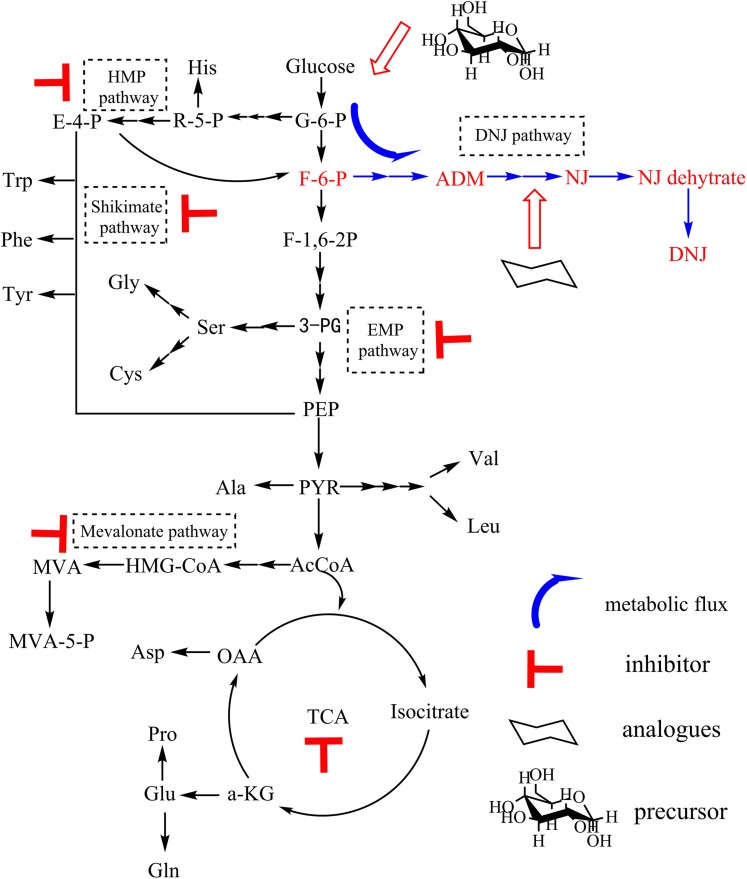
Strategies to enhance DNJ production based on its biosynthetic pathway.

The production of DNJ in *S. lavendulae* needed to be further enhanced because of the relatively low initial titer (approximately 90 mg/L) following cultivation in BFM for 3 days (Figure 1). Therefore, a novel strategy to regulate the production of DNJ was first designed through precursor feeding and using metabolic inhibitors. A strategy diagram depicting a method to enhance DNJ production based on its biosynthetic pathway is illustrated in Figure 4.

### Effect of Feeding Metabolic Inhibitors on the Production of DNJ

**FIGURE 5:**
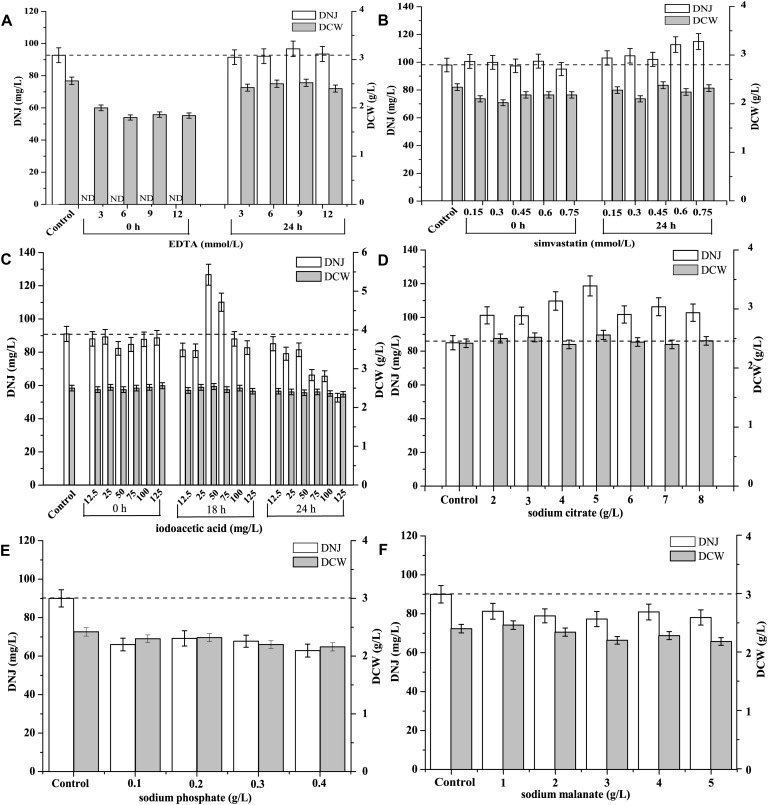
Effect of different metabolic inhibitors at different concentrations and various addition time on the production of DNJ and growth of UN-8. **(A)** EDTA, concentration was 3, 6, 9, 12 mmol/L and addition time was 0 and 24 h. **(B)** Simvastatin, concentration was 0.15, 0.30, 0.45, 0.60, 0.75 mmol/L and addition time was 0 and 24 h. **(C)** Iodoacetic acid, concentration was 12.5, 25, 50, 75, 100, 125 mmol/L and addition time was 0, 18 and 24 h. **(D)** Sodium citrate, concentration was 2, 3, 4, 5, 6, 7, 8 g/L and addition time was 0 h. **(E)** Sodium phosphate, concentration was 0.1, 0.2, 0.3, 0.4 g/L and addition time was 24 h. **(F)** Sodium malanate, concentration was 1, 2, 3, 4, 5 g/L and addition time was 24 h.

The effect of different concentrations of the shikimate pathway inhibitor EDTA on the production of DNJ and the growth of UN-8 is shown in Figure 5A. It was found that when EDTA (3–12 mmol/L) was added during the initial stage (0 h), the production of DNJ was inhibited during the whole fermentation process, and the growth of UN-8 was also slightly suppressed in contrast with the control group. When EDTA was added at the pre-synthesis stage of DNJ (24 h), the growth of UN-8 was not affected, and EDTA at 9 mmol/L could slightly increase the production of DNJ by approximately 5%, while other concentrations of EDTA did not stimulate the production of DNJ.

The effect of the mevalonate pathway inhibitor simvastatin on DNJ synthesis at different time is shown in Figure 5B. There was no obvious promotion of DNJ synthesis and UN-8 growth when different concentrations of simvastatin (0.15–0.75 mmol/L) were added at the initial stage (0 h). When the addition time was changed to 24 h, all the tested concentrations of simvastatin could facilitate the production of DNJ to some extent. However, when the concentration of simvastatin was 0.6 and 0.75 mmol/L, the DNJ titer was increased by 15.3 and 17.3%, respectively.

The effect of the EMP pathway inhibitor iodoacetic acid on the production of DNJ is presented in Figure 5C. It was found that there was no positive effect on the titer of DNJ when iodoacetic acid (12.5–125 mg/L) was added at 0 and 24 h. Some higher concentrations (24 h, 75–125 mg/L) inhibited the production of DNJ compared with the control group. However, when the addition time was changed to 18 h, iodoacetic acid (50 and 75 mg/L) could facilitate the synthesis of DNJ significantly, and the titer of DNJ was increased by 38.5 and 20.9%, respectively (Figure 5C).

The effects of another EMP pathway inhibitor, sodium citrate, on the titer of DNJ are shown in Figure 5D. When sodium citrate (2–8 g/L) was added at 0 h, it was found that all different tested concentrations could facilitate the production of DNJ. In particular, when the concentration of sodium citrate was 5 g/L, the titer of DNJ was increased by 39.5%, indicating that sodium citrate was an effective metabolic regulatory inhibitor for DNJ production. Additionally, sodium citrate did not promote the production of DNJ when the addition time was 24 h (data not shown).

Figure 5E demonstrates the effects of the HMP inhibitor sodium phosphate on the synthesis of DNJ. It can be seen that sodium phosphate (0.1–0.4 g/L) which was added at 24 h did not improve the titer of DNJ. On the contrary, the titer of DNJ decreased when compared with the control group. If the addition time was 0 h, the growth of UN-8 was inhibited markedly and the titer of DNJ was very low (data not shown).

Sodium malonate is a TCA inhibitor. Its effect on the titer of DNJ is shown in Figure 5F. It was evident that the addition of different concentrations of sodium malonate (1–5 g/L) at 24 h did not accelerate the production of DNJ. On the contrary, the addition of sodium malonate decreased the titer of DNJ, which was similar to the effect of sodium phosphate. The growth of UN-8 was also inhibited by sodium phosphate (1–5 g/L) added at 0 h (data not shown), suggesting that sodium malonate is not suitable as a regulatory inhibitor to control the production of DNJ.

### Effect of Feeding Precursor and Intermediate Analogs on the Production of DNJ

**FIGURE 6:**
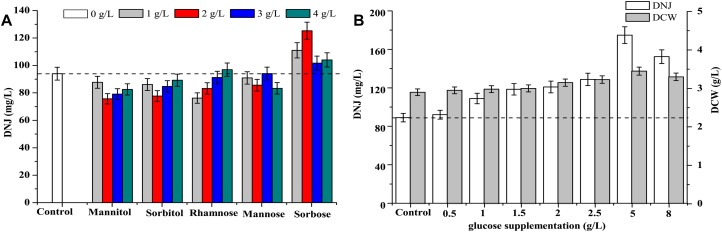
**(A)** Effect of addition of precursors and intermediate analogs on the synthesis of DNJ. **(B)** Effect of glucose supplementation on the synthesis of DNJ.

The effects of feeding precursor analogs, such as mannose (MW: 180.16) and sorbose (MW: 180.16) and intermediate analogs, such as mannitol (MW: 182.17), sorbitol (MW: 182.17), and rhamnose (MW: 164.16) on the titer of DNJ are presented in Figure 6A. The structures of these analogs are shown in Supplementary Figure S3. The results showed that only the addition of the precursor analog sorbose at 1, 2, 3, and 4 g/L at 0 h could enhance the production of DNJ by 17.2, 37.2, 7.8, and 10.2%, respectively. Other analogs such as mannitol, sorbitol, rhamnose, and mannose did not have a significant influence on the production of DNJ.

### Effect of Feeding Precursor Glucose on the Production of DNJ

As shown in Figure 1A, the residual glucose was completely consumed at 24 h, which might affect the titer of DNJ in the subsequent fermentation because the synthesis of DNJ relies on the existence of carbon sources (Stein et al., 1984). Therefore, the effects of feeding precursor glucose at 24 h on the production of DNJ were assessed and are illustrated in Figure 6B. It was clear that the titer of DNJ was improved with the addition of different concentrations of glucose (0.5–8 g/L). In particular, the titer of DNJ was raised by 96.4% when the concentration of added glucose was 5 g/L. Moreover, the biomass of UN-8 was also slightly increased with increasing concentrations of glucose, indicating that feeding precursor glucose was beneficial for the production of DNJ in *S. lavendulae*.

### Strategy Designed for the Production of DNJ Through Orthogonal L_27_(3^5^) Test Design

When the experimental factor, n, is approximately 5–13, the design L_27_(3^*n*^) can be selected as an orthogonal test design (Menon et al., 2013), using Minitab 17 Statistical software. The results of the orthogonal test design are demonstrated in Table 1. The maximum titer of DNJ reached 296.56 mg/L, which occurred in experiment 24. However, determining the best conditions for DNJ production required further orthogonal analysis. Therefore, the values of K and R were calculated and are displayed in Table 1, and the variance of the orthogonal experiments are listed in Table 2. The significance analysis suggested that all the tested factors, such as the concentration of supplemented glucose (A, *p* < 0.01), the time of glucose supplementation (B, *p* < 0.01), the concentration of sorbose (C, *p* < 0.01), and the time of iodoacetic acid supplementation (D, *p* < 0.01), had a significant influence on the titer of DNJ, which is consistent with the single factor experiment results. According to the R value, the degree of influence was in the order A (52.6 mg/L) > C (44.3 mg/L) > B (43.5 mg/L) > D (34.2 mg/L). Based on the higher K value at respective levels (A, level 3, 231.2 mg/L; B, level 2, 217.2 mg/L; C, level 1, 223.5 mg/L; D, level 3, 220.4 mg/L), the optimal orthogonal group was determined as A_3_B_2_C_1_D_3_. This group was in agreement with the highest titer of DNJ in experiment 24, as demonstrated in Table 1. The time course curves of DNJ content, UN-8 biomass, and residual glucose under the optimized fermentation strategy are also shown in Supplementary Figure S2. These results demonstrated that UN-8 grew well and its growth was maintained for a longer time (>3 g/L before 72 h) after glucose supplementation when compared with non-optimized medium (Figure 1A, <3 g/L after 42 h), indicating that good growth is beneficial for the production of DNJ. Overall, the strategy designed for the production of DNJ based on its biosynthetic pathway could be briefly described as follows. The initial fermentation medium (pH, 7.30 ± 0.10) was composed of yeast extract 10 g/L, glucose 5 g/L, sorbose 1 g/L, sodium citrate 5 g/L, NaCl 0.5 g/L, KNO_3_ 0.5 g/L, K_2_HPO_4_^∙^3H_2_O 0.5 g/L, MgSO_4_^∙^7H_2_O 0.5 g/L. After aerobic fermentation for 20 h, iodoacetic acid (50 mg/L) was added, and subsequently glucose (7 g/L) was also added at 26 h. Using this strategy, the titer of DNJ could reach 296.56 mg/L after 3 days, which was 3.3-fold higher than that of the non-optimized group (90 mg/L) fermented in BFM.

**TABLE 1:** The results of orthogonal experiment design.

**Run**	**A**	**B**	**C**	**D**	**E**	**DNJ (mg/mL)**
1	1	1	1	1	1	158.82
2	1	1	1	1	2	155.56
3	1	1	1	1	3	157.23
4	1	2	2	2	1	186.18
5	1	2	2	2	2	198.37
6	1	2	2	2	3	189.33
7	1	3	3	3	1	180.53
8	1	3	3	3	2	188.30
9	1	3	3	3	3	193.05
10	2	1	2	3	1	186.22
11	2	1	2	3	2	180.27
12	2	1	2	3	3	192.48
13	2	2	3	1	1	170.82.
14	2	2	3	1	2	172.29
15	2	2	3	1	3	175.22
16	2	3	1	2	1	227.95
17	2	3	1	2	2	225.73
18	2	3	1	2	3	223.71
19	3	1	3	2	1	181.82
20	3	1	3	2	2	172.40
21	3	1	3	2	3	178.62
22	3	2	1	3	1	280.14
23	3	2	1	3	2	286.06
24	3	2	1	3	3	296.56
25	3	3	2	1	1	228.71
26	3	3	2	1	2	225.90
27	3	3	2	1	3	230.95
K_1_	178.6	173.7	223.5	186.2	200.1	
K_2_	195.0	217.2	202.0	198.2	200.5	
K_3_	231.2	213.9	179.2	220.4	204.1	
R	52.6	43.5	44.3	34.2	4.0	

*A (concentration of glucose supplementation, g); B (time of glucose supplementation, h); C (concentration of sorbose addition at 0 h); D (time of iodoacetic acid addition, h, the concentration is 50 mg/L); E (error). K_1_ represented the average of nine mixed values of DNJ yields at the first level, and the same as K_2_ and K_3_; R was the result of extreme analysis.*

**TABLE 2:** Analysis of variance for orthogonal experimental.

**Factor**	**df**	**Adj SS**	**Adj MS**	***F* value**	**Significant**
Concentration of glucose supplementation	2	13064.9	6532.45	257.93	^∗∗^
Time of glucose supplementation	2	10550.3	5275.13	208.29	^∗∗^
Concentration of sorbose	2	8835.6	4417.82	174.44	^∗∗^
Time of iodoacetic acid supplementation	2	5426.8	2713.38	107.14	^∗∗^
Error	18	455.9	25.33		
Sum	26	38333.4			

*^∗∗^*p* < 0.01, extremely significant.*

## Discussion

As mentioned in the introduction, the DNJ-producing bacteria that have been discovered and reported so far are limited to *Bacillus* sp. and *Streptomyces* sp., such as *B. subtilis* (Stein et al., 1984), *B. amyloliquefaciens* (Cai et al., 2017), *S. lavendulae* (Wei et al., 2011), and *S. subrutilus* (Hardick et al., 1992). Although the biosynthetic pathway of DNJ is difficult to understand, previous studies in *B. subtilis* and *S. subrutilus* have provided an effective method to explore the pathway by precursor feeding combined with isotope-labeled experiments (Hardick et al., 1992; Hardick and Hutchinson, 1993).

Since secondary metabolites are often synthesized during the logarithmic growth phase or stationary phase of the microorganism, the addition of a carbon source at this stage could indicate whether it acted as a precursor or not. Therefore, assessing the time course of growth of UN-8 and the titer of DNJ was very important before conducting precursor feeding experiments (Figure 1).

In the current study, glucose was demonstrated to be a preferable precursor for the synthesis of DNJ in *S. lavendulae* (Figure 1B), which is consistent with the DNJ-producing strain *S. subrutilus* (Hardick et al., 1992). [1-^13^C] and [2-^13^C] labeled glucose experiments demonstrated that DNJ was formed through a C2-N-C6 cyclization reaction, indicating that the carbon skeleton of glucose underwent inversion during the DNJ synthesis process. This phenomenon was similar to that in *B. subtilis* and *S. subrutilus*, but different from that in *C. communis* in which DNJ was formed via a C1-N-C5 cyclization reaction (Shibano et al., 2004). The extraction results of endogenous cell components that were analyzed using a HILIC-MS/MS technique revealed the existence of metabolic intermediate compounds, such as ADM, NJ and its dehydrate, for the first time in *S. lavendulae* (Figure 2). NJ could be directly formed from ADM through an oxidation step (Figure 3). The structure and molecular weight of the three compounds were very similar to those of DNJ (Figure 2). Interestingly, an NJ epimer named mannojirimycin (MJ) and a DNJ epimer named 1-deoxymannojirimycin (DMJ) were not detected in cell extracts of *S. lavendulae* using the HILIC-MS/MS technique, indicating that MJ may not participate in the synthesis of DNJ. In contrast, MJ is an important intermediate in *S. subrutilus*, because it is the direct oxidation product of ADM (Hardick et al., 1992). Then, a portion of the MJ is converted to NJ through an epimerization step, yielding DNJ, while another portion of the MJ is used to synthesize DMJ. Taking this information into account, it was concluded that the biosynthetic pathway for DNJ in *S. lavendulae* is related to the glycolysis pathway, and that the pathway showed characteristics which are different from those in *S. subrutilus* and *B. subtilis*.

However, Kang et al. (2011) reported a putative operon involved in DNJ synthesis in *B. subtilis* MORI 3K-85. These genes were named *gabT1*, *yktC1*, and *gutB1* and were predicted to encode a putative transaminase, phosphatase, and oxidoreductase, respectively. However, to date, there have been no reports concerning the synthesis gene cluster of DNJ in *Streptomyces* sp. Some work was conducted in our research group, which involved attempting to clone the synthesis genes that are responsible for encoding putative transaminase, phosphatase, and oxidoreductase in *S. lavendulae* based on the *gabT1*, *yktC1*, and *gutB1* sequence information from *Bacillus* sp. However, we did not obtain positive results, and further work is required in this regard.

The initial titer of DNJ in *S. lavendulae* was somewhat low (approximately 90 mg/L) when it was cultivated in BFM (Figure 1A). Therefore, this titer needed to be improved. Based on the clarified biosynthetic pathway of DNJ presented above, a novel strategy was designed to enhance the production of DNJ by feeding synthetic precursors and specific metabolic inhibitors (Figure 4). Feeding metabolic inhibitors of rate-limiting enzymes would reduce carbon flux to other non-DNJ producing metabolite pathways, thereby facilitating the production of DNJ (Figure 5). The shikimate pathway starts from 4-phosphoric acid erythrose and phosphoenolpyruvate. It was reported that EDTA is an inhibitor for 3-dehydroquinate synthase or shikimate kinase (Sun et al., 2009). Inhibition of these two enzymes would slow down the carbon flux. Therefore, EDTA (9 mmol/L) added at 24 h slightly increased the production of DNJ by approximately 5% (Figure 5A). The rate-limiting enzyme of the mevalonate pathway is 3-Hydroxy-3-methylglutaryl-CoA reductase (HMGR). A statin compound such as simvastatin is a competitive inhibitor for HMGR, which catalyzes the conversion of 3-HMG-CoA to mevalonic acid (Buhaescu and Izzedine, 2007). Therefore, the titer of DNJ was enhanced by 17.3% with the addition of simvastatin (0.75 mmol/L) (Figure 5B). Iodoacetic acid and sodium citrate are known EMP inhibitors for glyceraldehyde-3-phosphate dehydrogenase and phosphofructokinase/pyruvate kinase, respectively (Wang et al., 2002; Liu et al., 2004). The addition of iodoacetic acid (50 mg/L) and sodium citrate (5 g/L) facilitated the synthesis of DNJ significantly, increasing it by 38.5 and 39.5%, respectively (Figures 5C,D). It has been reported that sodium phosphate can inhibit the activity of glucose-6-phosphate dehydrogenase, which catalyzes the conversion of glucose-6-phosphate to phosphogluconolactone (Yu and Pan, 1996). However, the reduced titer of DNJ suggested that HMP pathway regulation was not useful in increasing the production of DNJ (Figure 5E). The HMP pathway can provide reducing power through molecules such as NADPH, which is important for many enzymatic reactions (Wang et al., 2002). Taking the results of the metabolic inhibitor feeding experiments into consideration, it was obvious that the addition of EMP inhibitors (iodoacetic acid and sodium citrate) was more effective than inhibitors of the shikimate pathway, mevalonate pathway, HMP pathway, and TCA inhibitors, in increasing the production of DNJ, which may be linked to the fact that DNJ synthesis in *S. lavendulae* is closely related to the glycolysis pathway (Figure 4).

A previous study suggested that DNJ levels were highly dependent on carbon sources (Stein et al., 1984). Carbon sources not only provide the energy needed for microbial metabolism, but also provide an important carbon skeleton for the synthesis of compounds. Therefore, feeding precursors and intermediate analogs were used to accelerate the production of DNJ (Figure 6). In *B. amyloliquefaciens*, the addition of the intermediate analog sorbitol could enhance the production of DNJ (Onose et al., 2013). However, in the current study in *S. lavendulae*, the addition of sorbitol did not make any difference in this context (Figure 6A). On the contrary, the addition of the precursor analog sorbose (2 g/L) increased the production of DNJ by 37.2% (Figure 6A), which has not been reported in other DNJ-producing strains. Furthermore, no significant promotion of the DNJ titer was observed in *B. amyloliquefaciens* when supplemented with precursor glucose (Onose et al., 2013). Interestingly, galactose and lactose were verified as the best fermentation carbon sources for DNJ production in *B. subtilis* (Cho et al., 2008) and *B. amyloliquefaciens* (Yamagishi et al., 2017), and *Streptomyces* sp. (Paek et al., 1997), respectively. In contrast, the titer of DNJ was increased by 96.4% with the addition of precursor glucose (5 g/L) in the current study (Figure 6B), indicating that the addition of precursor glucose was very effective for DNJ production in *S. lavendulae*. This difference may be related to the carbon utilization characteristics of the microorganism itself, such as glucose utilization. A previous study revealed that a lower glucose concentration (5 g/L) was suitable for the growth of *S. lavendulae* UN-8. Once the concentration of glucose reached 40 g/L, the growth of UN-8 was completely inhibited (Wu et al., 2017). However, the DNJ-producing strain *B. amyloliquefaciens* could grow and produce DNJ well at 50 g/L of glucose. Taking the single factor experiment results into consideration, an orthogonal test design L_27_(3^5^) was used to optimize the production of DNJ. Finally, the titer of DNJ under these optimized conditions reached 296.56 mg/L, which was 3.3-fold higher than that of the control group (90 mg/L), suggesting that a regulation strategy based on the clarified biosynthetic pathway of DNJ was effective.

In summary, in the current study, the biosynthetic pathway of DNJ in *S. lavendulae* was characterized through precursor analysis, metabolic intermediate identification, and isotope-labeled D-[1-^13^C] and -[2-^13^C] glucose experiments. Glucose was confirmed to be a preferable precursor for DNJ synthesis. The biosynthetic pathway was related to the glycolysis pathway, and it started from fructose-6-phosphate, and involved amination, dephosphorylation, oxidation, cyclization, dehydration, and reduction reaction steps, yielding DNJ. Moreover, based on a strategy that exploited this biosynthetic pathway information, the titer of DNJ was greatly increased by adding sodium citrate (0 h, 5 g/L), sorbose (0 h, 1 g/L), iodoacetic acid (20 h, 50 mg/L), and glucose (26 h, 7 g/L) during the fermentation process, which provides new insight into the regulation of other secondary metabolites. To the best of our knowledge, this is the first report concerning the regulation of DNJ production from a biosynthetic pathway perspective.

## Data Availability

All datasets generated for this study are included in the manuscript and the Supplementary Files.

## Author Contributions

HW wrote the manuscript and performed the experiments. YG, LC, and GC analyzed the data. ZL conceived and designed the study. All authors discussed, read, and approved the final manuscript.

## Conflict of Interest Statement

The authors declare that the research was conducted in the absence of any commercial or financial relationships that could be construed as a potential conflict of interest.
