# Supplementary figures and images for: A Novel Strategy to Regulate 1-Deoxynojirimycin Production Based on Its Biosynthetic Pathway in Streptomyces lavendulae
Source: Front Microbiol. 2019 Aug 22;10:1968. doi: 10.3389/fmicb.2019.01968 (PMC6713920; doi:10.3389/fmicb.2019.01968)

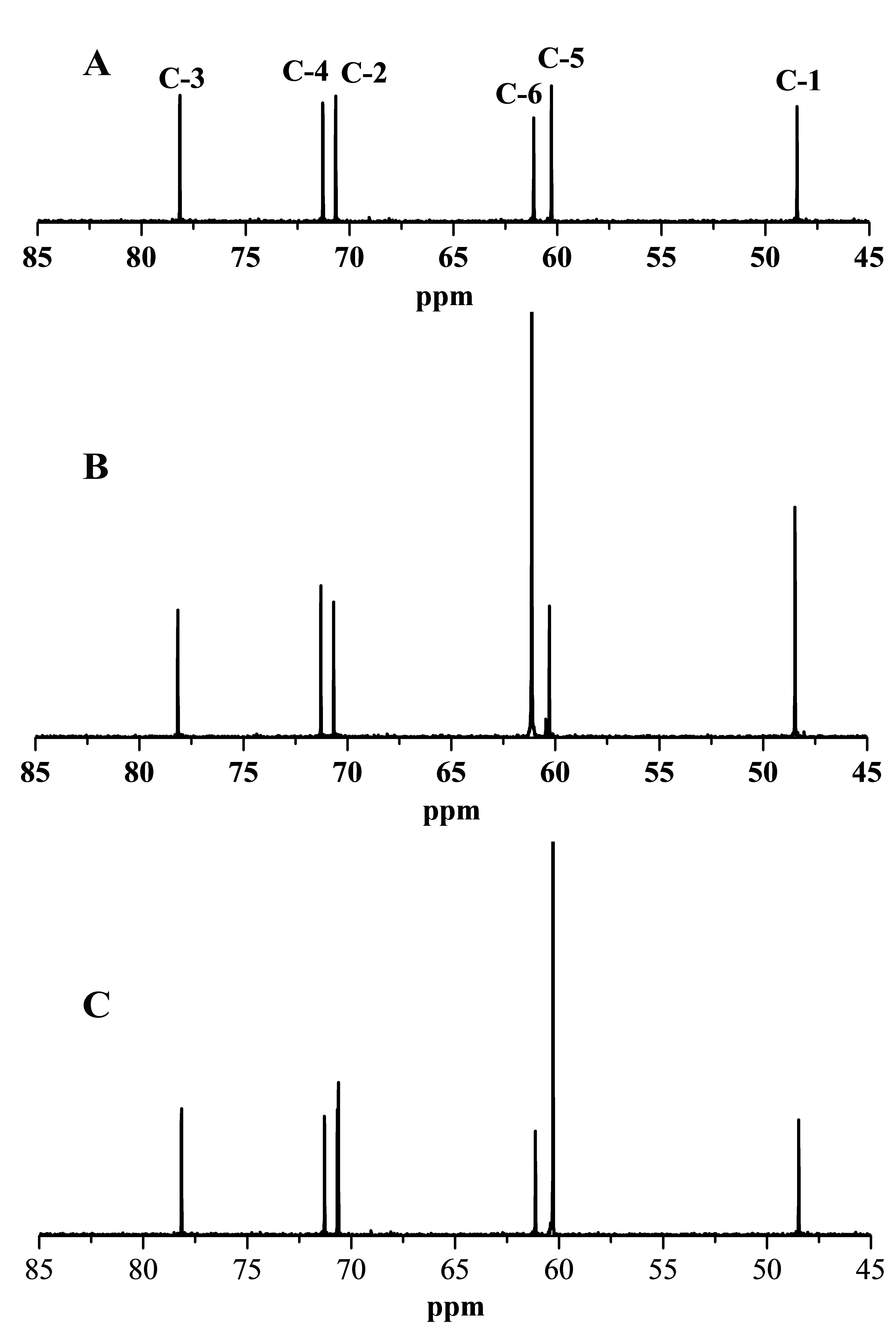

Supplement: Supplementary file 1 [file Image_1.TIF]
