# Supplementary material for: A Novel Strategy to Regulate 1-Deoxynojirimycin Production Based on Its Biosynthetic Pathway in Streptomyces lavendulae
Source: Front Microbiol. 2019 Aug 22;10:1968. doi: 10.3389/fmicb.2019.01968 (PMC6713920; doi:10.3389/fmicb.2019.01968)
Supplement: Supplementary file 4 [file Table_1.DOCX]

Supplemental material for Frontiers in Microbiology

**Fig. S1** NMR spectrum of DNJ. (**A**), DNJ synthesized with unlabeled glucose as precursor; (**B**), DNJ synthesized with labeled 1-^13^C-glucose as precursor; (**C**), DNJ synthesized with labeled 2-^13^C-glucose as precursor

**Fig. S2** Time course curves of DNJ content, biomass of UN-8, residual glucose under the optimized fermentation strategy

**Fig. S3** The chemical structures of mannose, sorbose, mannitol, sorbitol, rhamnose

**Table S1**. Different regulation strategies of adding metabolism inhibitors, precursor and intermediate analogs on the production of DNJ

| Regulation strategies | Compounds | Concentrations | Time | Target enzyme |
| --- | --- | --- | --- | --- |
| Mevalonate pathway inhibitor | simvastatin | 0.15-0.75 mM | 0 h, 24 h | HMGR (Buhaescu and Izzedine, 2007) |
| Shikimate pathway inhibitor | EDTA | 3-12 mM | 0 h, 24 h | DHQS, SK (Sun et al., 2009) |
| EMP inhibitors | iodoacetic acid | 12.5-125 mg/L | 0 h, 18 h, 24 h | GAPDH (Wang et al., 2002) |
|  | sodium citrate | 2-8 g/L | 0 h | PFK, PK (Liu et al., 2004) |
| HMP inhibitor | sodium phosphate | 0.1-0.4 g/L | 24 h | G6PD (Yu and Pan, 1996) |
| TCA inhibitor | sodium malonate | 1-5 g/L | 24 h | SDH (Zeng et al., 2019) |
| Precursor and intermediate analogs | mannitol, sorbitol, rhamnose, mannose, sorbose | 1-4 g/L | 0 h |  |
| Precursor | glucose | 0.5-8 g/L | 24 h |  |

HMGR, 3-Hydroxy-3-methylglutaryl-CoA reductase; DHQS, 3-Dehydroquinate synthase; SK, shikimate kinase;

GAPDH, glyceraldehyde-3-phosphate dehydrogenase; PFK, phosphofructokinase; PK, pyruvate kinase

G6PD, Glucose-6-phosphate dehydrogenase; SDH, succinodehydrogenase;

**Table S2**. Factors and levels for the orthogonal test in 250 ml shake flask

| Variable | level | | |
| --- | --- | --- | --- |
|  | 1 | 2 | 3 |
| A, Concentration of glucose supplementation (g) | 0.250 | 0.300 | 0.350 |
| B, Time of glucose supplementation (h) | 24 | 26 | 28 |
| C, Concentration of sorbose (g) | 0.050 | 0.075 | 0.100 |
| D, Time of iodoacetic acid supplementation (h) | 16 | 18 | 20 |
| E, Error |  |  |  |
